# Three-Dimensional Ankle Moments and Nonlinear Summation of Rat Triceps Surae Muscles

**DOI:** 10.1371/journal.pone.0111595

**Published:** 2014-10-31

**Authors:** Chris Tijs, Jaap H. van Dieën, Guus C. Baan, Huub Maas

**Affiliations:** MOVE Research Institute Amsterdam, Faculty of Human Movement Sciences, VU University Amsterdam, Amsterdam, The Netherlands; Emory University School Of Medicine, United States of America

## Abstract

The Achilles tendon and epimuscular connective tissues mechanically link the triceps surae muscles. These pathways may cause joint moments exerted by each muscle individually not to sum linearly, both in magnitude and direction. The aims were (i) to assess effects of sagittal plane ankle angle (varied between 150° and 70°) on isometric ankle moments, in both magnitude and direction, exerted by active rat triceps surae muscles, (ii) to assess ankle moment summation between those muscles for a range of ankle angles and (iii) to assess effects of sagittal plane ankle angle and muscle activation on Achilles tendon length. At each ankle angle, soleus (SO) and gastrocnemius (GA) muscles were first excited separately to assess ankle-angle moment characteristics and subsequently both muscles were excited simultaneously to investigate moment summation. The magnitude of ankle moment exerted by SO and GA, the SO direction in the transverse and sagittal planes, and the GA direction in the transverse plane were significantly affected by ankle angle. SO moment direction in the frontal and sagittal planes were significantly different from that of GA. Nonlinear magnitude summation varied between 0.6±2.9% and −3.6±2.9%, while the nonlinear direction summation varied between 0.3±0.4° and −0.4±0.7° in the transverse plane, between 0.5±0.4° and 0.1±0.4° in the frontal plane, and between 3.0±7.9° and 0.3±2.3° in the sagittal plane. Changes in tendon length caused by SO contraction were significantly lower than those during contraction of GA and GA+SO simultaneously. Thus, moments exerted by GA and SO sum nonlinearly both in the magnitude and direction. The limited degree of nonlinear summation may be explained by different mechanisms acting in opposite directions.

## Introduction

The triceps surae muscles are essential for locomotion in both quadrupedal and bipedal animals. Soleus (SO), medial (MG) and lateral (LG) gastrocnemius muscles are co-activated during human gait [1] and cycling [2], as well as during rat [3] and cat [4] locomotion. In comparison, selective activation of these muscles has been observed during prolonged, low-level static plantar-flexion in humans [5] and during paw-shakes in cats [6]. It is well known that, when these muscles are active, they exert a plantar-flexion moment at the ankle joint. In addition, ankle moments outside the sagittal plane of individual triceps surae muscles were found in cats [7], [8], and, more recently, MG in humans was suggested to contribute to body stabilization in the frontal plane [9]. Despite the fact that the mechanical interaction of animals with the environment during several movement tasks is not restricted to the sagittal plane, ankle moments outside the sagittal plane have received much less attention.

Musculoskeletal models can be used to predict 3D joint moments exerted by individual muscles. These models assume single attachment sites for the muscle origin and insertion. Inaccuracies might be introduced when muscles in fact have origin and/or insertion sites distributed along the skeleton [10]. These models also assume muscles to act as independent actuators [11], an assumption that is also applied when using functional electrical stimulation (FES) to control limb movements. However, the triceps surae distal tendons merge into the Achilles tendon. This common elastic element mechanically connects the triceps surae muscles to each other. As a consequence, length changes of the Achilles tendon will affect the muscle belly lengths of all triceps surae muscles and, hence, their force production. Furthermore, it has been shown that connective tissue linkages between adjacent muscles are capable of transmitting muscle force [12], [13]. The extent of such epimuscular myofascial force transmission has been shown to be dependent on the relative position of muscle bellies [14]. Thus, mechanical interactions between triceps surae muscles can occur via two pathways: (i) the Achilles tendon and (ii) epimuscular myofascial connections.

The functional relevance of the pathways of intermuscular mechanical interaction is still unclear. To test this, the joint moment exerted by simultaneous excitation of the triceps surae muscles can be compared with the mathematical sum of the moment exerted when each muscle is excited individually. If these pathways are relevant for intermuscular mechanical interactions, the triceps surae muscles cannot be regarded as independent actuators and the sum of the joint moments exerted by each muscle individually will not be equal to the joint moment during simultaneous excitation of these muscles, which is commonly referred to as nonlinear summation [15]. This indicates that the moment each of these muscles exert is altered due to co-contraction with the other muscle. End-point forces measured at the distal part of the tibia on excitation of different combinations of rat upper hindlimb muscles did sum nonlinearly [16]. Ankle moment summation of MG and the combined LG+SO complex in cats was also found to be nonlinear [17]. Both studies reported rather small values of nonlinear summation (<10%), but these values were found for only one position of the hindlimb. To date, it is unknown whether nonlinear summation is joint angle dependent. For the triceps surae muscle group, nonlinear summation may increase at more plantar-flexed ankle positions, most likely because this involves lower muscle-tendon-unit lengths and, hence, lower stiffness of the common elastic Achilles tendon.

Therefore, the aims of the present study were (i) to assess effects of sagittal plane ankle angle on isometric ankle moments, in both magnitude and direction, exerted by active rat triceps surae muscles, (ii) to assess ankle moment summation between the LG+MG complex (GA) and SO muscle for a range of ankle angles and (iii) to assess effects of sagittal plane ankle angle and muscle activation on Achilles tendon length.

## Materials and Methods

### Animals

For this study, 12 male Wistar rats (body mass: 309.5±10.3 g, mean±s.d.) were used. Surgical and experimental procedures were in agreement with the guidelines and regulations concerning animal welfare and experimentation set forth by Dutch law, and approved by the Committee on Ethics of Animal Experimentation at the VU University Amsterdam (Permit Number: FBW 11-02). All animals were euthanized at the end of the experiment with an overdose of intracardially-injected pentobarbital sodium followed by a double-sided pneumothorax.

Rats were anesthetized using intraperitoneally injected urethane (initial dose 1.2 ml/100 g body mass, 12.5% urethane solution). Deepness of anesthesia was tested throughout the experiment by evaluating if palpebral reflexes, ear withdrawal reflexes and hindlimb withdrawal reflexes could be elicited. Supplemental doses (0.3–0.5 ml) of urethane were given as needed, to suppress all of the above reflexes. To prevent hypothermia during surgery and data collection, the animals were placed on an electrical heating pad, maintaining core temperature at approximately 37°C. Frequently applying saline solution prevented dehydration of nerves, muscles and connective tissues.

### Surgery

The left hindlimb was shaved and the skin and biceps femoris muscle were removed. Anatomical landmarks for axis of rotation of ankle (medial and lateral malleoli) and knee joints (origin of medial and lateral collateral ligament) were identified and marked with black and white ink. The femur was exposed for attachment of a clamp. Tissues between the malleoli and Achilles tendon were removed to secure the calcaneus to the set-up. To identify length changes of the portion of the Achilles tendon shared by GA and SO, a marker was placed on its posterior side at the point where the tendons of both muscles merge (∼45% of the LG distal tendon length).

**Figure 1:**
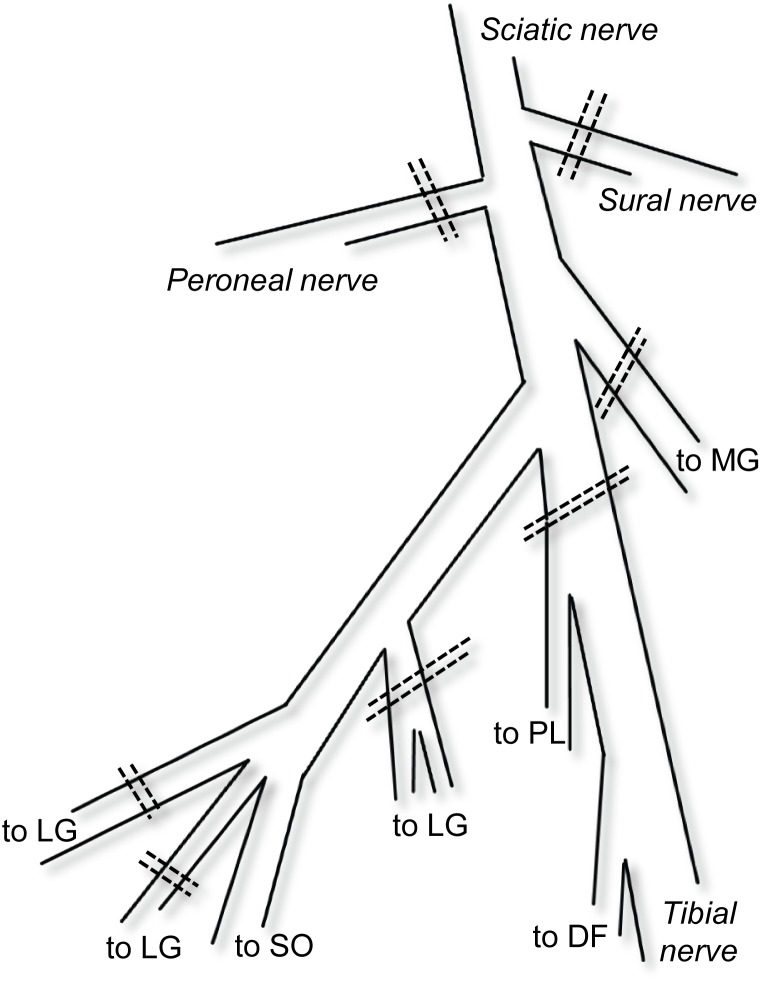
Schematic view of branches of the sciatic nerve in the popliteal fossa. To excite only SO muscle fibers, a cuff electrode was applied onto the sciatic nerve and several nerve branches were transected. Dashed lines represent transection of nerves. MG: medial gastrocnemius. LG: lateral gastrocnemius. SO: soleus. PL: plantaris. DF: deep flexor muscles.

The sciatic nerve was partly dissected free for placement of a cuff electrode. To disrupt neural connections between the muscles and the central nervous system, the sciatic nerve was crushed proximal to the cuff electrode. In the popliteal fossa, the sciatic nerve divides into the peroneal nerve, the sural nerve and the tibial nerve (Fig. 1). The peroneal and sural nerves, as well as the MG nerve branch were cut directly at their bifurcation. The tibial nerve was cut distal to the SO+LG nerve branch. To access the SO+LG nerve branch, proximal MG and LG muscle bellies were separated by ∼5–10 millimetres. However, SO muscle and its epimuscular myofascial connections with LG and plantaris (PL) muscle were not affected by this procedure. Individual nerve branches for LG muscle (typically 3 or 4) were identified using stimulation with a bipolar hook electrode and cut. Thus, only the branch innervating SO muscle was kept intact.

MG and LG muscles consist of multiple compartments with multiple motor endplates [18], [19]. Bipolar intramuscular wire electrodes were inserted near motor endplates located in the distal region of MG and the proximal region of LG.

### Fixation in experimental apparatus

**Figure 2:**
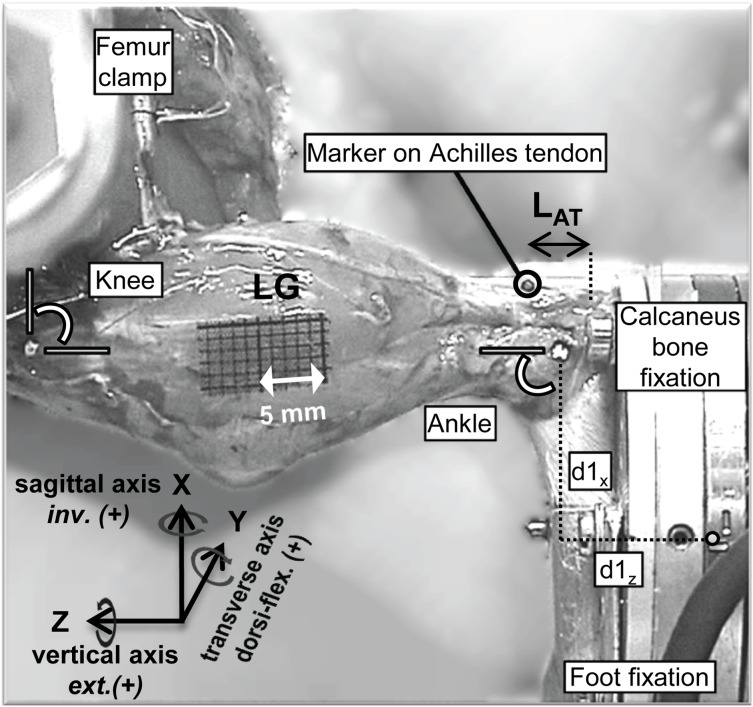
Lateral view of the rat left hindlimb in the set-up. Ankle and knee joints were at 90° around the transverse axis. The femur was fixed and the foot was attached to the load cell using custom-made clamps. Anatomical landmarks for ankle and knee joint were aligned with the set-up’s rotational axes. A marker was placed on the posterior side of the Achilles tendon, which was used to assess length (L_AT_) and length changes of the distal part of the Achilles tendon. Positive ankle moments around the sagittal, transverse and vertical axis indicate inversion (inv.), dorsi-flexion (dorsi-flex.) and external rotation (ext.), respectively and are parallel to the x-, y- and z-axis of the load cell. In addition, d1_x_ and d1_z_ represent the distance from the center of the load cell to the center of the ankle joint in the x- and z-direction, respectively. LG: lateral gastrocnemius. Dotted vertical line represents the insertion site of the Achilles tendon.

The left hindlimb was secured to the experimental set-up by clamping the femur and attaching the foot to a 6 degrees-of-freedom load cell (Mini40-E, ATI, Apex, NC, USA). For alignment of the ankle and knee joint with the set-up’s rotational axes, knee and ankle joints were set to an included angle (i.e. the smallest angle between the tibia and the foot) of 90° around the transverse axis (Fig. 2). Custom clamps were used to secure the dorsal surface of the foot and the calcaneus to the load cell. The midpoint between both malleoli was aligned with the origin of the y-axis of the load cell. In addition, the midpoint between the bilateral landmarks of the knee joint was aligned with the midpoint between both malleoli using a laser pointer. We defined the position of the axes of the knee and ankle joint as a line through the medial epicondyle of the femur and medial malleolus of the tibia, respectively, both perpendicular to the sagittal plane (i.e. parallel to the y-axis of the load cell). The position of the joint axes was assumed to be constant. Finally, the x- and z-position of the medial anatomical landmarks of ankle (d1_x_ and d1_z_, see Fig. 2 and eq. 3) and knee joint were aligned with the set-up’s rotational axes and their position relative to the origin of the load cell was measured.

### Experimental protocol

SO muscle was maximally excited by supramaximal stimulation of the sciatic nerve via the bipolar cuff electrode connected to a constant current source (0.4–0.5 mA). MG and LG muscles were simultaneously excited (0.7–2.0 mA) via bipolar intramuscular fine wire electrodes inserted near their motor endplates. As the threshold current near a motor endplate is substantially lower than direct excitation of muscle fibers [20], the chance of excitation of surrounding muscles was minimized. To determine stimulation amplitude, the current was increased from threshold current up to an amplitude in which an increase in stimulation amplitude did not result in an increase in active ankle moment. As only one MG and one LG muscle compartment were stimulated, not all muscle fibers were excited. One pilot experiment was performed in which LG+MG were excited simultaneously via nerve stimulation and via intramuscular stimulation. Peak active LG+MG ankle moment during intramuscular stimulation (27.4 mNm) was substantially lower than that during nerve stimulation (144.9 mNm). This indicates that 18.9% of maximum active ankle moment exerted by MG and LG muscles fibers was exerted when stimulated intramuscularly.

**Figure 3:**
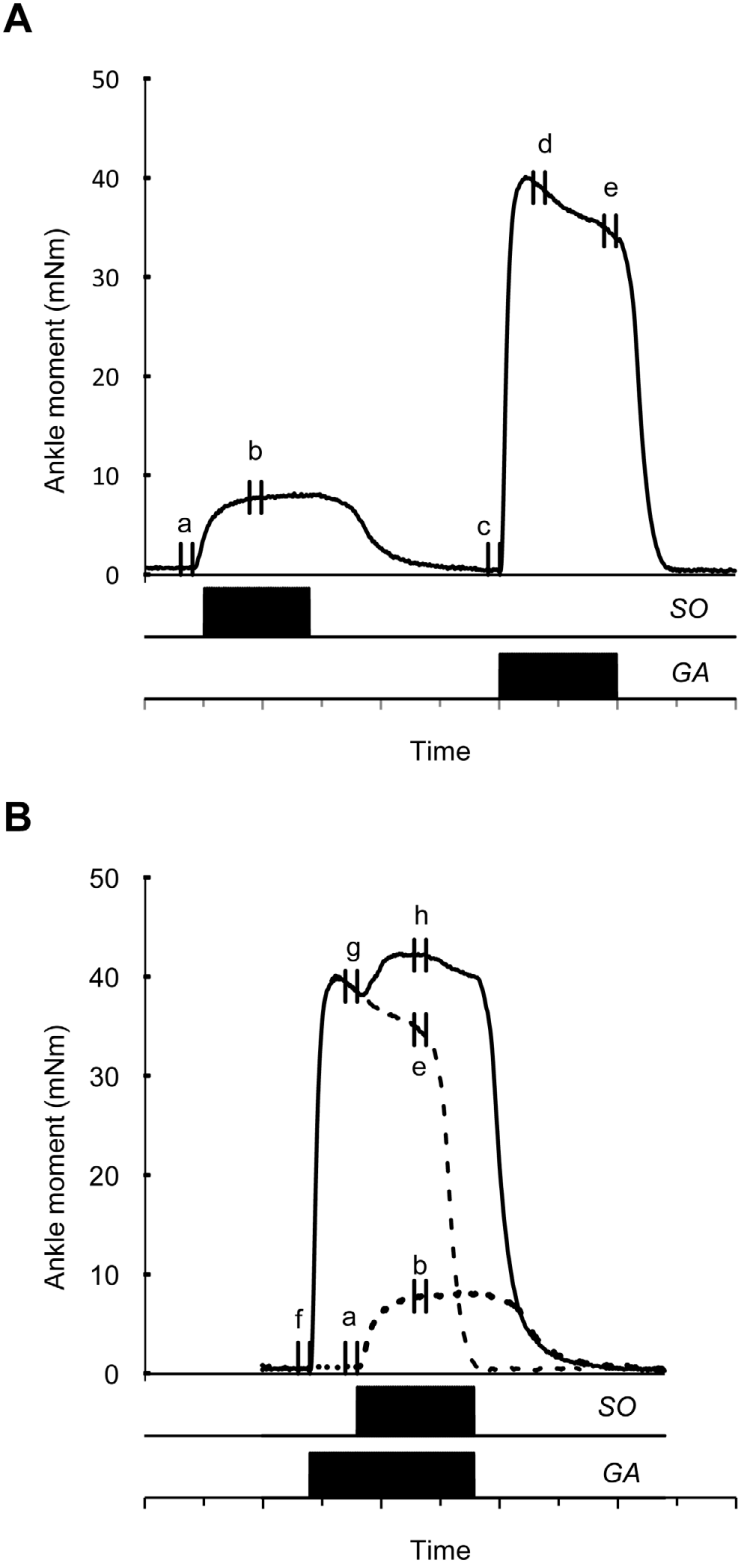
Schematic view of the two stimulation protocols. In this example, ankle joint was positioned at 95°. A) Stimulation (500 ms) of SO and GA separately. B) Simultaneous stimulation (solid line) of GA (700 ms) and SO (500 ms). Dotted and dashed lines represent the individual SO and GA ankle moments as shown in Fig. 3A, respectively. Ankle moments were assessed for several time windows (a–h) by calculating the mean for each 50-ms time windows (see Data analysis).

In each experiment, the ankle angle was varied only around the transverse axis (i.e., plantar-flexion/dorsi-flexion): from 150° to 90° with steps of 5° and from 90° to 70° with steps of 10°. Steps of 5° were used because (i) large changes in ankle moments were expected and, therefore, more data points were deemed necessary for an accurate assessment of the moment-angle curve and (ii) determination of the optimum ankle angle can be done with more precision if smaller angle steps are used. Beyond optimum, steps of 10° were used to limit the number of contractions, which especially at high muscle lengths can result in tissue damage. The knee joint was kept constant at 90° around the transverse axis. Both ankle and knee joint were kept at 0° around the other axes. Note that changing the ankle from a plantar-flexed angle (150°) to a dorsi-flexed angle (70°) results in lengthening of all triceps surae muscles. For each angle of the ankle joint, two stimulation protocols were performed (Fig. 3). First, isometric ankle moments were assessed during tetanic contraction (100 Hz, 500 ms) of either SO or GA (LG+MG) muscles (Fig. 3A). Second, isometric ankle moments were assessed during tetanic contractions of both GA and SO (Fig. 3B), but with trains of different length (i.e., GA was excited for 700 ms followed after 200 ms by SO for 500 ms). After each stimulation protocol, two minutes rest periods with the ankle at 105° were allowed. Both stimulation protocols were performed consecutively for each ankle angle. Therefore, potential effects of fatigue due to an increasing number of muscle contractions did not affect assessment of nonlinear moment summation. Prior to and after testing all ankle angles, control measurements were performed at 150° and 120° to monitor any changes in muscle conditions (e.g., fatigue) that could affect SO and GA ankle angle-moment characteristics. Video recordings (SONY, DRC-TRV20E, 720×576 pixels, 25 frames/s, resolution 1 pixel ∼0.1 mm) were made to record Achilles tendon marker position before and during isometric muscle contractions.

### Data analysis

Forces (F’_x_, F’_y_, F’_z_) and moments (M’_x_, M’_y_, M’_z_) measured by the load cell were corrected for gravity caused by the mass of the load cell itself and the additional plates attached for foot fixation. Ankle moments (M_ankle_) were calculated around three axes (Fig. 2; M_x_: inversion(+)/eversion, M_y_: dorsi-flexion(+)/plantar-flexion, M_z_: external rotation(+)/internal rotation):
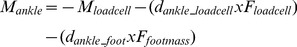
(1)where M_loadcell_ represents the moments measured by the center of the load cell; d_ankle_loadcell_ the distance from the center of the ankle position to the center of the load cell; F_loadcell_ the forces measured by the center of the load cell; d_ankle_foot_ the distance from the center of the ankle position to the center of mass (CoM) of the foot; and F_footmass_ the force due to the foot mass (1.7±0.1 g, *n = 3*), which is dependent on the orientation of the loadcell (α). For each animal, d1_x_ and d1_z_ were measured (Fig. 2), while d1_y_ was zero. A fixed distance between the position of the ankle and CoM of the foot ([21]; details anatomical data via personal communication Wehner) was assumed (eq. 3).



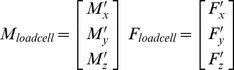
(2)

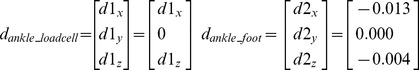
(3)

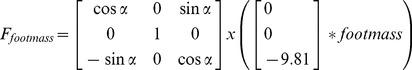
(4)


For each ankle angle, ankle moments in each direction were assessed before (passive) and during muscle contractions (total) by calculating the mean for 50-ms time windows (Fig. 3). Active ankle moments were calculated by subtracting the passive moment from the total moment at equal ankle joint angles. Time windows ‘*b*’ and ‘*e*’ were used to assess active ankle moments for SO (*b-a*) and GA (*e-c*), respectively. Time window ‘*h*’ was used to access the active ankle moments in each direction during simultaneous GA+SO excitation (*h-f)*. In addition, the difference in active GA ankle moments between time window ‘*g*’ and ‘*d*’ (*g-d*) was calculated for each ankle angle and for each direction (average eversion moment: 0.06±0.14 mNm; plantar-flexion moment: 0.2±0.7 mNm; internal rotation moment: −0.02±0.15 mNm). These values were subtracted from the active ankle moment exerted by GA+SO to exclude possible effects of initial differences in GA muscle excitation on nonlinear summation.

**Figure 4:**
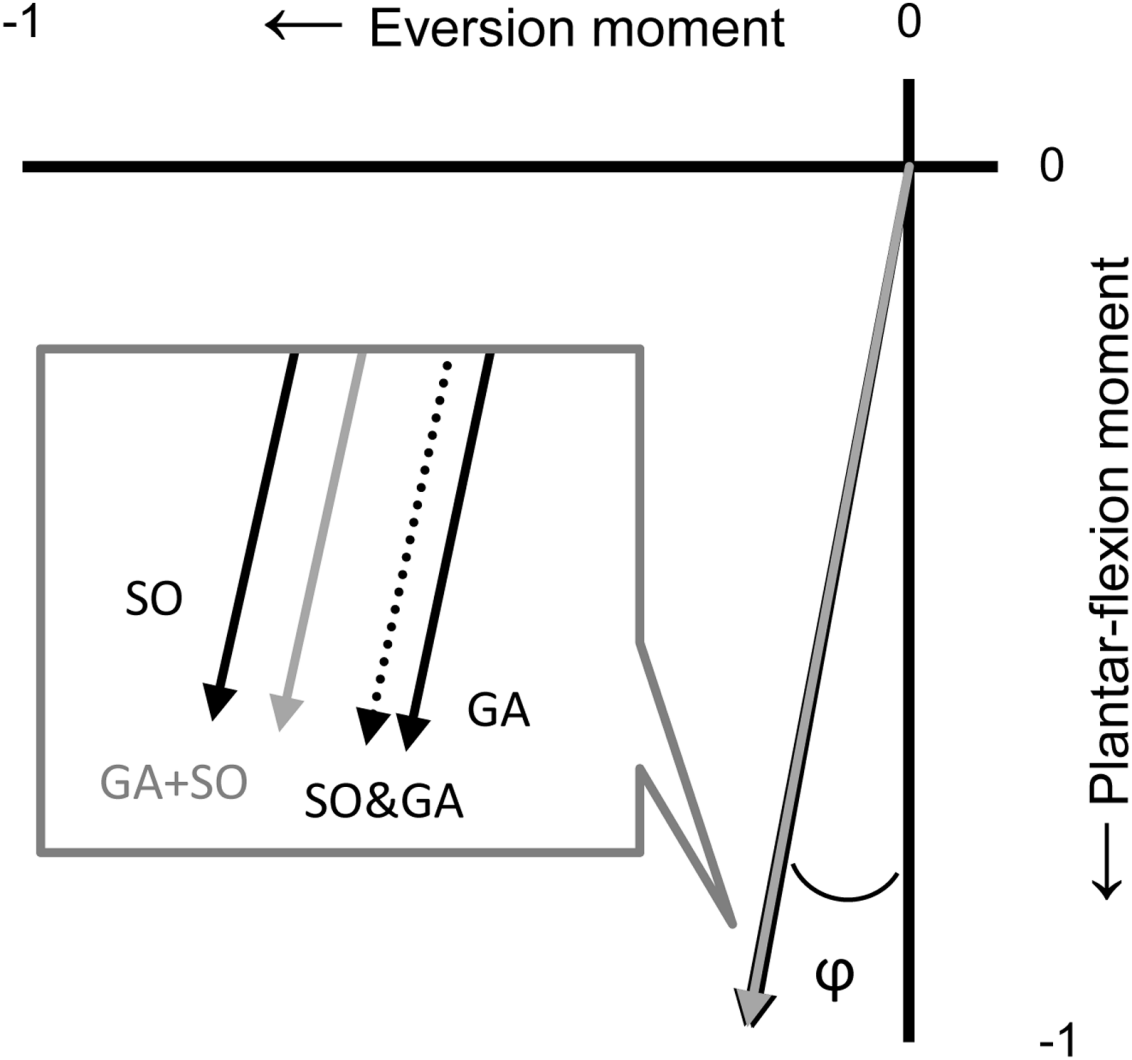
Unit vector direction of the ankle moments in the transverse plane. The unit vectors of SO (black solid line), GA (black solid line), the mathematical sum of SO&GA (black dotted line) and the simultaneously excitation of GA+SO (grey solid line) at 100° ankle angle. Knee angle was kept constant at 90°. For each vector in the transverse and frontal planes, the angle relative to the plantar-flexion moment axis was calculated (see φ), while for each vector in the sagittal plane, the angle relative to the eversion moment axis was calculated. These angles were used as a measure of the vector direction (see Fig. 6).



The active ankle moments in each direction were used to calculate the magnitude of the 3D ankle moment vector (eq. 5) and to calculate the direction of the vector in the three anatomical planes for the individual excitation of SO and GA, and for the simultaneous GA+SO excitation. For each vector in the transverse and frontal planes, the angle relative to the plantar-flexion moment axis was calculated (see φ in Fig. 4), while for each vector in the sagittal plane, the angle relative to the eversion moment axis was calculated.

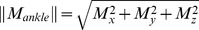
(5)Nonlinear magnitude summation was assessed by subtracting the 3D magnitude of SO and GA ankle moments from the 3D magnitude of GA+SO ankle moments (eq. 6), which was also normalized (%M_nl_) relative to the mathematical sum of the ankle moments exerted on excitation of SO and GA individually (eq. 7). Nonlinear direction summation was assessed by subtracting the mathematical sum of the vector direction during individual SO and GA excitation from the vector direction during GA+SO excitation.
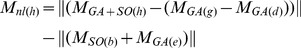
(6)

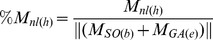
(7)


The length of the distal portion of the Achilles tendon was assessed by calculating the distance from the Achilles tendon insertion to the tendon marker (L_AT_ in Fig. 2). At high (i.e., plantar-flexed) ankle angles, the Achilles tendon is slack and therefore not a straight line. To prevent underestimation of its length, the curvature of the Achilles tendon was taken into account when calculating L_AT_. Achilles tendon marker displacement as a result of SO, GA and GA+SO contraction was calculated by subtracting the marker position before muscle contraction from the position during SO, GA and GA+SO muscle contractions.

The magnitude of the active 3D moment vector exerted by SO and GA muscles was calculated for the control measurements at 150° and 120° before and after the full experiment. All calculations were performed in MATLAB (R2011a, Mathworks, Natick, MA, USA).

### Statistics

One-way repeated measures ANOVA (SPSS Statistics 20, IBM Corporation, Armonk, NY, USA) with ‘ankle angle’ as independent factor (15 levels) was used to test for effects of ankle angle on the active SO and GA ankle moments in the magnitude and direction in the transverse, frontal and sagittal planes. A two-way repeated measures ANOVA with ‘ankle angle’ (15 levels) and ‘muscle’ (2 levels: SO and GA) as independent factors were used to test if the direction of the ankle moment exerted by GA was significantly different from SO. A one-sample t-test was used to test if the relative nonlinear magnitude summation (%M_nl_) and absolute amplitude of nonlinear direction summation averaged across ankle angles was significantly different from zero. A one-way repeated measures ANOVA with ‘ankle angle’ as independent factor (15 levels) was used to test if the relative nonlinear magnitude summation (%M_nl_) and nonlinear direction summation was affected by ankle angle. To test for effects of muscle contraction on Achilles tendon length changes, a two-way repeated measures ANOVA with ‘ankle angle’ (15 levels) and ‘stimulation’ (3 levels: SO, GA and GA+SO) as independent factors was used. Greenhouse-geisser correction was used when assumption of sphericity was violated. Level of significance was set at p<0.05. In two experiments, only SO muscle was excited, resulting in n = 12 for the analyses of SO ankle moments, while all other analyses included n = 10.

## Results

### Ankle angle-active moment characteristics of soleus muscle

**Figure 5:**
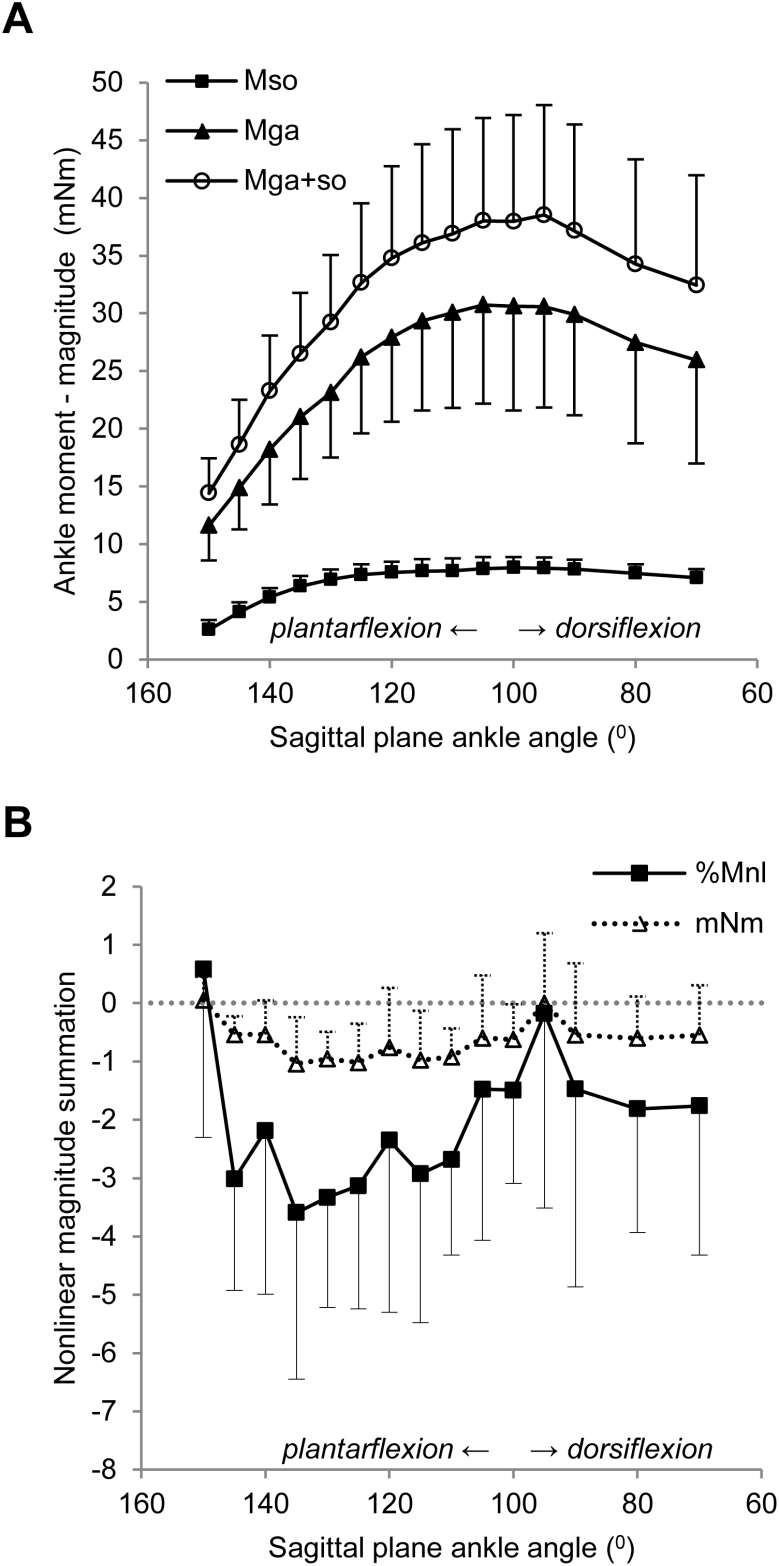
Effects of ankle dorsi-flexion on nonlinear moment summation of SO and GA muscles. A) Active 3D moment exerted by simultaneous excitation of GA+SO (○), and by individual excitation of SO (▪) and GA (▴) plotted as a function of sagittal plane ankle angle. Knee angle was kept constant at 90°. B) Effect of sagittal plane ankle angle on nonlinear magnitude summation, both in mNm (Mnl) and relative to the mathematical sum of the ankle moments exerted by individual SO and GA muscles (%Mnl). Negative values indicate a lower ankle moment exerted on simultaneous excitation of GA+SO muscles than the mathematical sum of the ankle moments exerted by individual SO and GA muscles. No effects of the sagittal plane ankle angle on relative nonlinear magnitude summation (p = 0.138) was found after excluding the data at 150° from the analysis. Averaged across all ankle angles except 150°, the relative nonlinear magnitude summation was significantly different from zero (p<0.001). Means ± s.d. are shown (n = 10).

**Figure 6:**
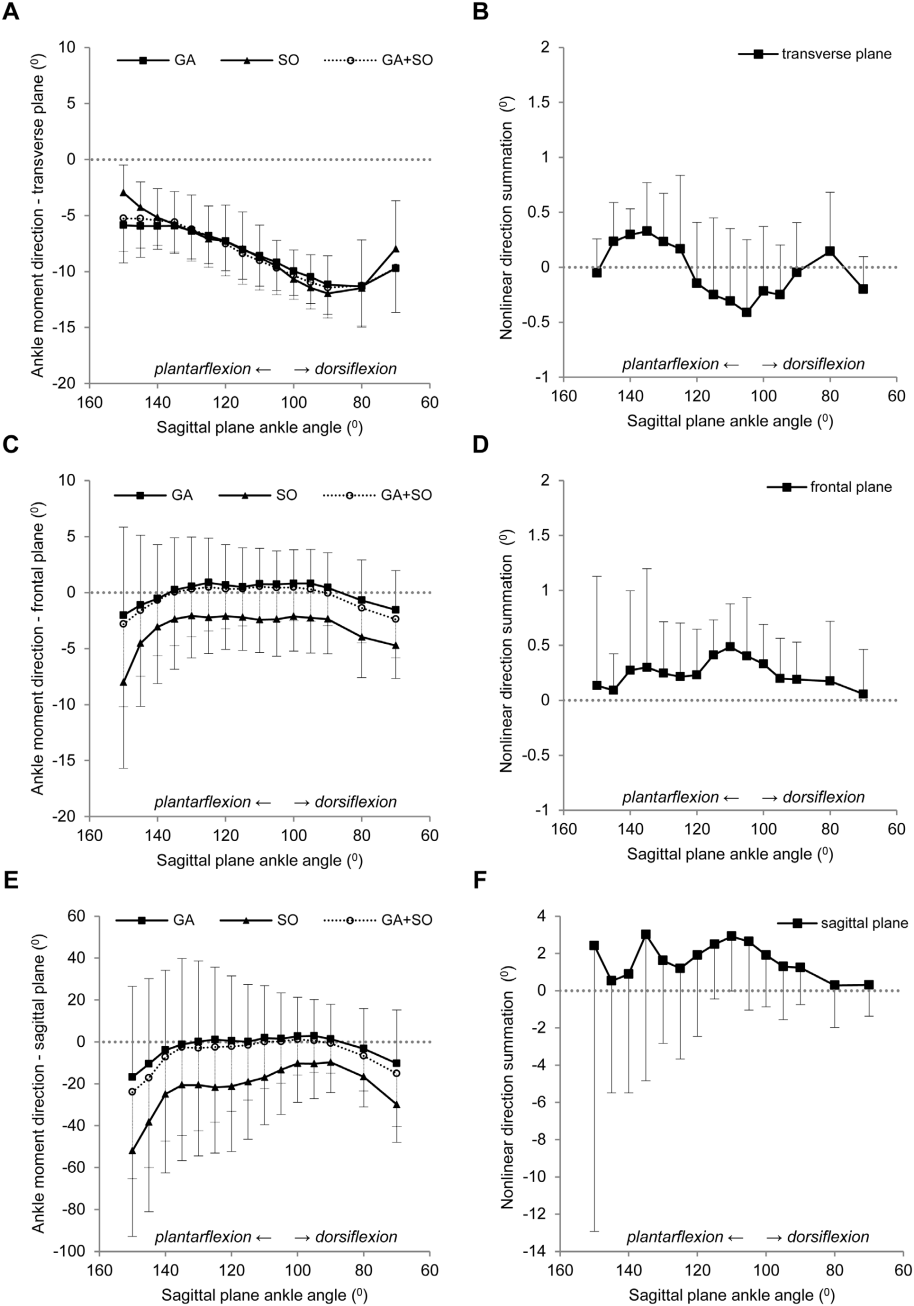
Effect of ankle dorsi-flexion on the nonlinear direction summation in the transverse, frontal and sagittal planes. Active moment direction in the transverse (A), frontal (C) and sagittal (E) planes exerted by simultaneous excitation of GA+SO (○), and by individual excitation of SO (▪) and GA (▴) plotted as a function of sagittal plane ankle angle. Knee angle was kept constant at 90°. Negative values indicate an eversion vector in the transverse plane and an internal rotation vector in the frontal and sagittal planes. Significant effects of ankle angle on the SO (p<0.001) and GA (p<0.001) vector in the transverse plane and on the SO vector (p = 0.021) in the sagittal plane were found. SO vector in the frontal plane (p = 0.059) and GA vectors in the frontal (p = 0.217) and sagittal (p = 0.245) planes were, however, not affected by ankle angle. The effect of sagittal plane ankle angle on the direction summation in the transverse, frontal and sagittal planes are presented in panel B, D and F, respectively. Ankle angle did not significantly affect the nonlinear direction summation in the transverse (p = 0.078), frontal (p = 0.684) and sagittal (p = 0.820) planes. The absolute amplitude of nonlinear direction summation was, however, on average significantly different from zero in all planes (p<0.001). Means ± s.d. are shown (n = 10).

Ankle dorsi-flexion, causing an increase in SO muscle-tendon-unit length, changed the magnitude of the active SO ankle moment (p<0.001). SO ankle moment increased up to 100° ankle angle to a peak moment of 8.0±0.9 mNm (Fig. 5A). Thereafter, the ankle moment decreased, but only by 0.9±0.6 mNm. Ankle dorsi-flexion also changed the direction of the SO ankle moment vector in the transverse plane (p<0.001) from −3.0±2.5° at 150° to a peak angle of −12.0±3.4° at 90° (Fig. 6A) and in the sagittal plane (p = 0.021) from −52.0±40.9° at 150° to a minimum angle of −9.8±14.5° at 90° (Fig. 6E). In the frontal plane, the average direction was −3.1±4.2° (Fig. 6C), which was not significantly affected by ankle angle (p = 0.059). Control measurements revealed no changes in active SO moments due to previous muscle contractions (p = 0.716), which indicates that SO muscle was not fatigued. These results show that SO muscle exerts moments in all three directions, but only the direction in the transverse and sagittal planes was affected by changes in sagittal plane ankle angle.

### Ankle angle-active moment characteristics of gastrocnemius muscle

Similar to SO muscle, ankle dorsi-flexion affected the magnitude of the active GA ankle moment (p<0.001). GA ankle moment increased from 11.6±3.0 mNm at 150° to a peak moment of mNm at 105° (Fig. 5A). The direction of the GA ankle moment vector in the transverse plane was also affected by ankle angle (p<0.001) and ranged from −5.9±3.4° at 150° to −11.3±3.6° at 80° (Fig. 6A), which was not significantly different from SO muscle (p = 0.870). The direction of the GA ankle moment in the frontal (mean: 0.0±4.2°, Fig. 6C) and sagittal (mean: −2.3±29.7°, Fig. 6E) planes, however, were not affected by ankle angle (p = 0.217 and p = 0.245, respectively), although they were significantly different from SO muscle (p = 0.001 for both planes). In contrast to SO, control measurements revealed evidence of fatigue in GA muscle. Previous muscle contractions decreased the 3D moment significantly: by –10.2±5.9% at 150° (p = 0.001) and by −12.1±10.1% at 120° (p = 0.008). As the ankle angles were always tested from the most plantar-flexed to the most dorsi-flexed angle, GA active moments of the more dorsi-flexed ankle angles were thus somewhat underestimated. Nonetheless, the results indicate that GA muscle exerted not only substantial plantar-flexion moment, but also an eversion moment, and that the direction of the ankle moment in the transverse plane was significantly affected by changes in sagittal plane ankle angle.

### Nonlinear moment summation of SO and GA muscle moments

Nonlinear magnitude summation ranged from 0.6±2.9% (0.1±0.4 mNm) at 150° to −3.6±2.9% (–1.0±0.8 mNm) at 135° (Fig. 5B). ANOVA indicated a significant effect of sagittal plane ankle angle on the nonlinear magnitude summation (p = 0.013). However, this effect was absent (p = 0.138) after excluding the data at 150° from the analysis, suggesting no effects of ankle angle on the nonlinear magnitude summation for the majority of ankle angles. Averaged across all ankle angles except 150°, the nonlinear magnitude moment summation was −0.7±0.8 mNm (Eqn. 6), that is −2.2±2.6% of the mathematical sum (Fig. 5B, Eqn. 7), which was significantly different from zero (p<0.001).

Nonlinear direction summation ranged from 0.3±0.4° at 135° to −0.4±0.7° at 105° in the transverse plane (Fig. 6B), from 0.5±0.4° at 110° to 0.1±0.4° at 70° in the frontal plane, (Fig. 6D) and from 3.0±7.9° at 135° to 0.3±2.3° at 80° in the sagittal plane (Fig. 6F). ANOVA indicated no significant effect of sagittal plane ankle angle on the values of nonlinear direction summation in the transverse (p = 0.078), frontal (p = 0.684) and sagittal (p = 0.820) planes. However, averaged across ankle angles, the absolute amplitude of nonlinear direction summation was greater than zero in the transverse (on average: 0.4±0.3°, p<0.001), frontal (on average: 0.4±0.4°, p<0.001) and sagittal plane (on average: 3.4±4.7°, p<0.001). These results indicate that both the magnitude of the moments exerted by SO and GA and the direction of its vector do not sum linearly.

### Achilles tendon length changes

**Figure 7:**
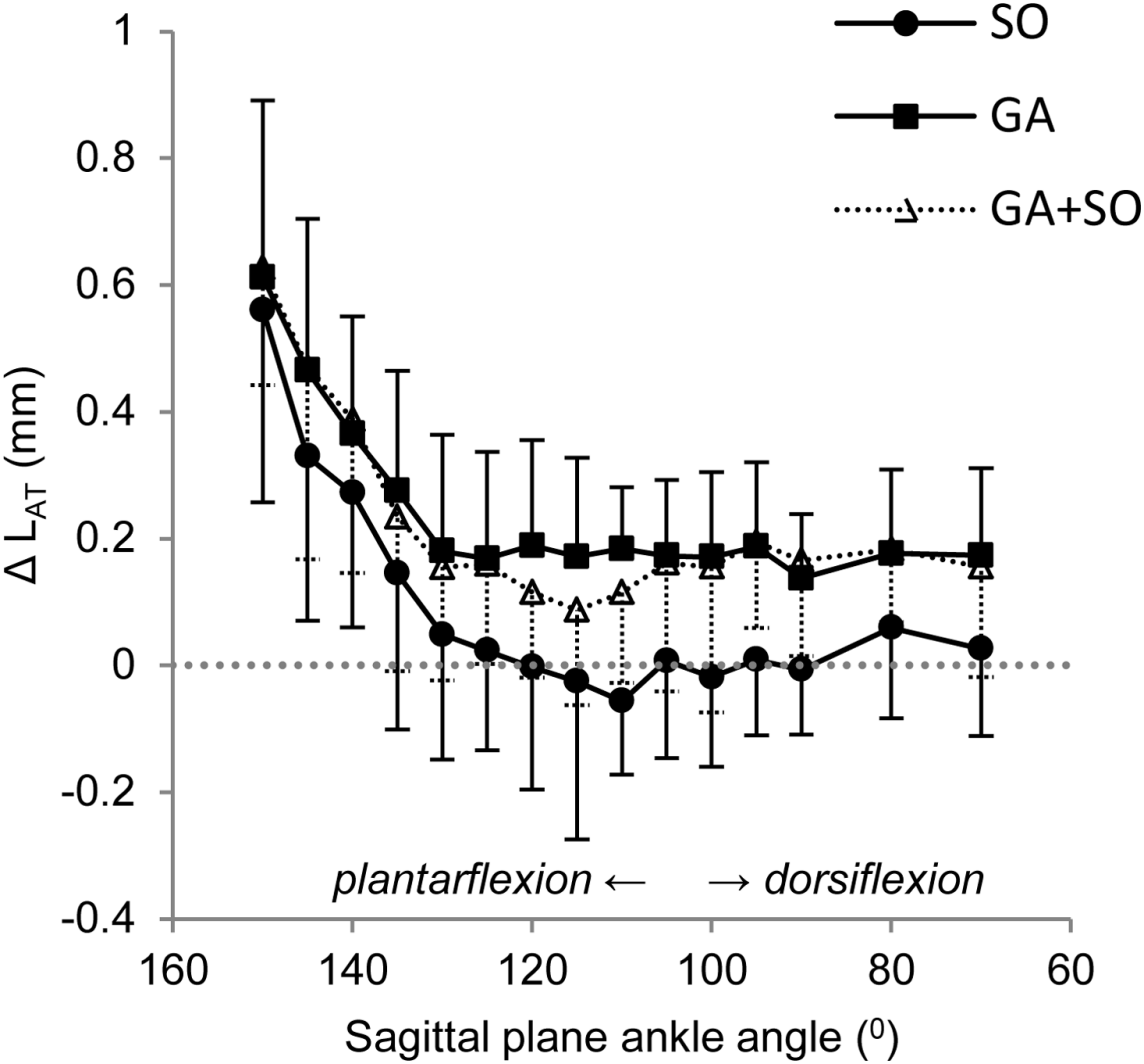
Effect of muscle activation on the length changes of the shared distal portion of the Achilles tendon. Changes in Achilles tendon length (ΔL_AT_) in response to simultaneous contraction of GA+SO (Δ), and to individual contraction of SO (•) and GA (▪) are plotted as a function of sagittal plane ankle angle. Knee angle was kept constant at 90°. Effects of ankle angle were found for SO, GA and GA+SO (p<0.001). Length changes due to SO contraction were lower than length changes due to GA (p = 0.033) and GA+SO (p = 0.028) contraction. No differences were found between GA and GA+SO contractions (p = 1.000). Means ± s.d. are shown (n = 10).

We found that length changes in response to SO, GA, and GA+SO muscle contractions (Fig. 7) were significantly affected by ankle angle (p<0.001). Changes in length caused by SO contraction were significantly lower than those during contraction of GA (p = 0.033) and GA+SO (p = 0.028). However, no significant differences were found between GA and GA+SO contractions (p = 1.000). In addition, no significant interaction effects were found (p = 0.148). Achilles tendon length changes in response to muscle contraction were highest at the most plantar-flexed ankle angle (i.e., lowest triceps surae muscle length). Changes in Achilles tendon length were rather constant between 130° and 70°, being 0.17±0.14 mm and 0.15±0.13 mm in response to GA and GA+SO contraction, respectively, but negligible (0.01±0.16 mm) in response to SO contraction. These results indicate that the distal portion of Achilles tendon was lengthened more during simultaneous GA+SO contraction than during contraction of SO exclusively.

## Discussion

This is the first study that investigated active SO and GA ankle moments in all three dimensions, as well as ankle moment summation of SO and GA muscles, for an extensive range of sagittal plane ankle angles in rats. We showed that varying the sagittal plane ankle angle affected the magnitude of the ankle moments exerted by SO and GA muscles, the direction of SO in the transverse and sagittal planes and the direction of GA in the transverse plane. We also showed that, in the frontal and sagittal planes, the direction of the SO vector was significantly different from the direction of the GA vector. Finally, the magnitude of the ankle moment exerted by SO and GA muscles and the direction of their vectors in the transverse, frontal and sagittal planes did not sum linearly. In contrast to our hypothesis, nonlinear magnitude summation was not dependent on sagittal plane ankle angle.

### Active ankle angle-moment characteristics of SO and GA muscles

Our results showed that SO and GA muscles were not pure plantar-flexors, but that they also exerted substantial eversion moments (i.e. direction of the vectors of both muscles in the transverse plane were not 0°). This can be explained by the lateral insertion of the rat Achilles tendon on the calcaneus [11]. In humans, both an inversion moment arm [22] and an inversion moment [9] for the MG muscle have been reported. In cats, SO moment arms have only been described for the plantar-flexion/dorsi-flexion direction [23] and have been assumed to be zero in the other directions [24]. However, ankle moments outside the sagittal plane have been reported for SO muscle in cats [8]. In contrast to our results, substantial external rotation but much smaller eversion moments were reported for cat MG and LG muscles [7], [8], their magnitudes being dependent on the 3D ankle angle [25]. The external rotation moment of cat LG and MG decreased when the ankle was rotated from an externally rotated angle to an internally rotated angle, as well as from an inverted to an everted angle. The external rotation moment found for cat MG and LG was likely the result of an externally rotated and/or inverted angle of the ankle joint. Because the external-internal angle and inversion-eversion angle in our study were close to zero, the different results can most likely be explained by differences in the 3D angle at which the ankle joint was secured in the setup.

### Ankle moment summation of SO and GA muscles

We found that the moments of SO and GA muscles sum nonlinearly, although the magnitude (ranged from 0.6% to −3.6%) of nonlinear summation was limited. Several mechanisms can cause nonlinear magnitude summation: (i) An increase in Achilles tendon moment arm in response to GA contraction as reported for humans [26]. Video recordings in our study did indeed show that the Achilles tendon moment arm in the sagittal plane increased (by up to ∼12%) in response to GA contraction (data not shown). Substantial changes were found only at the most plantar-flexed ankle angles (150–135 deg.). At these ankle angles, the Achilles tendon is likely slack prior to and pulled straight during muscle activation. In contrast with our findings, this would cause the SO ankle moment to be higher during additional GA activation than when GA muscle is inactive, leading to superadditive moment summation (i.e., Mga+so > Mso + Mga). Thus, other mechanisms are required to explain the subadditive moment summation found.

(ii) As suggested earlier [15], stretch of common elastic components, such as the Achilles tendon, can cause nonlinear force summation. Shortening of human GA muscle by passive knee flexion not only results in Achilles tendon shortening, but also in lengthening of SO muscle fascicles [27]. Such lengthening of SO muscle fascicles can be explained by mechanical interaction between GA and SO muscles via the Achilles tendon. Although strain within the Achilles tendon is not necessarily uniform [28], distal lengthening of the Achilles tendon during GA muscle contraction found in the present study (Fig. 7) most likely caused shortening of the SO muscle belly. As a consequence, SO force production when GA was activated simultaneously was different from SO contraction alone (the direction being dependent on the length of the SO muscle fibers with respect to optimum length). Note that, although we found negligible effects of SO activation on Achilles tendon length, with the limited pixel resolution (1 pixel ∼0.1 mm) we cannot exclude the possibility that also SO activation did affect the length of GA muscle fibers. Because GA ankle moments were 4–5 times higher than SO ankle moments (Fig. 5A), changes smaller than 0.1 mm will affect force exerted by GA. If Achilles tendon force increases its stiffness with ankle dorsi-flexion, effects of common elasticity on nonlinear magnitude summation were expected to be lower in more dorsi-flexed ankle angles. However, although nonlinear magnitude summation seemed highest at the more plantar-flexed ankle angles (except for 150°), we did not find a statistically significant angle effect. Note that, in the current study, the GA muscle was activated to only 18.9% of its maximum. Higher levels of muscle activation will result in higher forces exerted at the Achilles tendon and, consequently, greater nonlinear magnitude summation than observed.

(iii) It has also been shown that epimuscular myofascial connections between adjacent muscle bellies exist and that these connections are capable of transmitting muscle forces [12], [13]. The configuration of these connections and, hence, the magnitude of epimuscular myofascial force transmission, is influenced by the position of a muscle relative to its surroundings [29]. Changes of sagittal plane ankle angle while keeping the knee angle constant, as examined in the present study, does probably not result in significant changes of relative positions between GA and SO, since both muscles have similar insertion points on the calcaneus. As a consequence, the stiffness of epimuscular myofascial connections between these muscles was most likely not altered by the different experimental conditions. However, GA muscle contraction does result in shortening of its muscle belly and, thereby, changes the position of the GA muscle belly relative to that of SO to some extent. If GA muscle belly displacement affects SO force production, it will result in nonlinear magnitude summation. In a previous study no effects of knee angle on SO ankle moment were found [30], suggesting no myofascial interactions between passive GA and PL muscles with SO. However, activation of GA in our study may facilitate mechanical interaction via epimuscular myofascial connections [13] and, therefore, cannot be excluded as a mechanism contributing to nonlinear magnitude summation. Such effects may be enhanced at larger knee joint angles.

Nonlinear summation was found not only for the magnitude, but also for the direction in the transverse (up to 0.6°), frontal (up to 0.7°) and sagittal planes (up to 7.2°). Considering the different moment directions of SO compared to GA (Fig. 6C and Fig. 6E), a positive value of nonlinear direction summation for the frontal (Fig. 6D) and sagittal (Fig. 6F) planes indicates that the direction of the vector during simultaneous GA+SO excitation was more similar to that during individual GA excitation than predicted based on the mathematical sum. The higher forces exerted by GA compared to those of SO apparently changed the direction of the SO vector towards that of GA. This may be explained by a change in direction of the Achilles tendon upon GA activation. Alternatively, during simultaneous excitation some SO muscle force may be transmitted to the skeleton via GA muscle belly and tendon. This suggests that during simultaneous activation of SO and GA, the direction of ankle moment in the frontal and sagittal planes is biased towards the muscle with the highest force.

To assess nonlinear summation, active muscle moments must be used [17], [31]. As active moments are calculated by subtracting the passive moment prior to contraction from the total moment, it is implicitly assumed that the passive moments before and during muscle contraction are equal. This assumption has been shown to be valid for conditions in which passive forces are low, i.e. on the ascending limb of the length-force curve [32]. Recently, it has been found that up to at least 90° of the ankle, keeping the knee joint kept at 90°, both GA and SO muscles in the rat are operating below their optimum length and passive forces are negligible [33]. By using the same 90° knee angle in the present study, passive forces were therefore likely to be minimal for most of the ankle angles tested. However, caution is warranted when applying this method at extremely dorsi-flexed ankle positions and more extended knee positions.

In summary, the mechanical actions of SO and GA muscle on the ankle joint are not only determined by their origin and insertion on the skeleton, but also by activation of synergistic muscles. Because the compartment containing triceps surae muscles was kept as intact as possible, the specific contribution of the Achilles tendon and effects of epimuscular myofascial connections to nonlinear moment summation cannot be distinguished in this study.

Only a few studies have investigated nonlinear moment summation between muscles. Ankle moments exerted by cat triceps surae muscles showed low magnitudes (1.9-2.3%) of subadditive summation [17], [34]. More recently, summation of end-point forces, measured at the distal part of the tibia, on different combinations of rat hindlimb muscles was investigated [16]. The mathematical sum of 2D endpoint forces of randomly chosen hindlimb muscles was not equal to the endpoint forces during simultaneous stimulation of the same muscles. R^2^ values of 0.91 (for x-direction) and 0.93 (for y-direction), as measures of the similarity between the mathematical sum and the actual force, were reported. Thus, the magnitude of nonlinear summation between muscles seems to be limited. The nonlinear magnitude summation (–2.2±2.6%) found in our study are, therefore, in agreement with previous results. Although the net moment exertion of SO and GA suggests that these muscles sum nearly linearly, both in magnitude and direction, it should be noted that the mechanisms described above can have opposite effects on the ankle moments resulting in a small net effect.

Understanding the mechanical effects of the triceps surae muscles is relevant not only for constructing and validating musculoskeletal models of the ankle joint, but also for practical applications such as FES. In existing models for various animal species, SO and GA muscles are represented as independent point-to-point actuators without a joined Achilles tendon (for human [35], cat [36], and rat [11]). Linear summation of the mechanical effect of multiple muscles is also an important assumption of FES systems [16], being a property simplifying its controllers. The data of the present study provide new information about the validity of such approaches. The functional consequence of the limited nonlinear moment summation is at present unclear, but it may be larger for tasks that require a more precise joint movement control (e.g., finger prehension) and less pronounced for gross motor tasks such as walking.

## Conclusions

In this study, triceps surae muscles were shown not to exert only a plantar-flexor moment at the ankle, but they also had a substantial eversion vector. This eversion vector was dependent on the sagittal plane ankle angle. We also found that the SO vector was more directed towards an internal rotation moment than the GA vector. Nonlinear ankle moment summation was found, both in the magnitude and in the direction in the transverse, frontal and sagittal planes. However, they were limited and not affected by sagittal plane ankle angle. We hypothesize that this is the result of opposite effects of different mechanisms that can affect the mechanical interaction between GA and SO muscles.

## Supporting Information

File S1 Supplementary tables containing individual data from which the summary data are presented in the manuscript. Individual data on body mass (sheet: Descriptives), nonlinear magnitude summation as presented in Figure 5 (sheet: Figure5), nonlinear direction summation as presented in Figure 6 (sheet: Figure6) and Achilles tendon lengthening upon muscle activation as presented in Figure 7 (sheet: Figure7).
(XLSX)
